# Data on the radioprotective effect of emodin *in vivo* and *vitro* via inhibition of apoptosis and modulation of p53

**DOI:** 10.1016/j.dib.2016.12.038

**Published:** 2016-12-30

**Authors:** Jing Wang, Yue Zhang, Qiuzhen Zhu, Yulan Liu, Hao Cheng, Yuefan Zhang, Tiejun Li

**Affiliations:** a Department of Pharmacology, College of Pharmacy, Second Military Medical University, Shanghai 200433, China; b College of Pharmacology, Anhui University of Chinese Medicine, Hefei, Anhui 230012, China

## Abstract

This paper contains the experiment data on the emodin, mice and cell survival rate, mice intestinal tissue H&E and TUNEL staining, the expression of p53 protein in mice small intestine, cell apoptosis, the expression of protein and RNA *in vitro* included. Data was worked out through MTT assay, Flow cytometry, Western blot, Real-time PCR and Staining.

**Specifications Table**

**Table:** 

Subject area	*Biology*
More specific subject area	*Radioprotective effect;Pharmacology*
Type of data	*Figure, pathology image, dot graph, band graph*
How data was required	*Flow cytometry, Microplate Reader, fluorescence staining*
Data format	*Analyzed*
Experimental factors	*Pretreatment with 15,30,60 mg/kg/day emodin for 7 days to mice before exposing to radiation*
Experiment features	*Analysis with tissue staining,Hoechst staining and Western blot in vivo*
Data source lacation	*Shanghai, China*
Data accessibility	*Data are provided with this article*

**Value of the data**

• Data show the radioprotective effect of emodin *in vivo*. It is never reported before.
• The importance of using morphological features based on symptoms of gastrointestinal acute radiation symptom can serve as a guideline to facilitate recognition and quantification of these radiation-induced gastrointestinal tract injury.
• p53 apoptosis signaling pathways are the probable molecular mechanism of emodin radioprotective effect.

## 1 Data

**Fig. 1:**
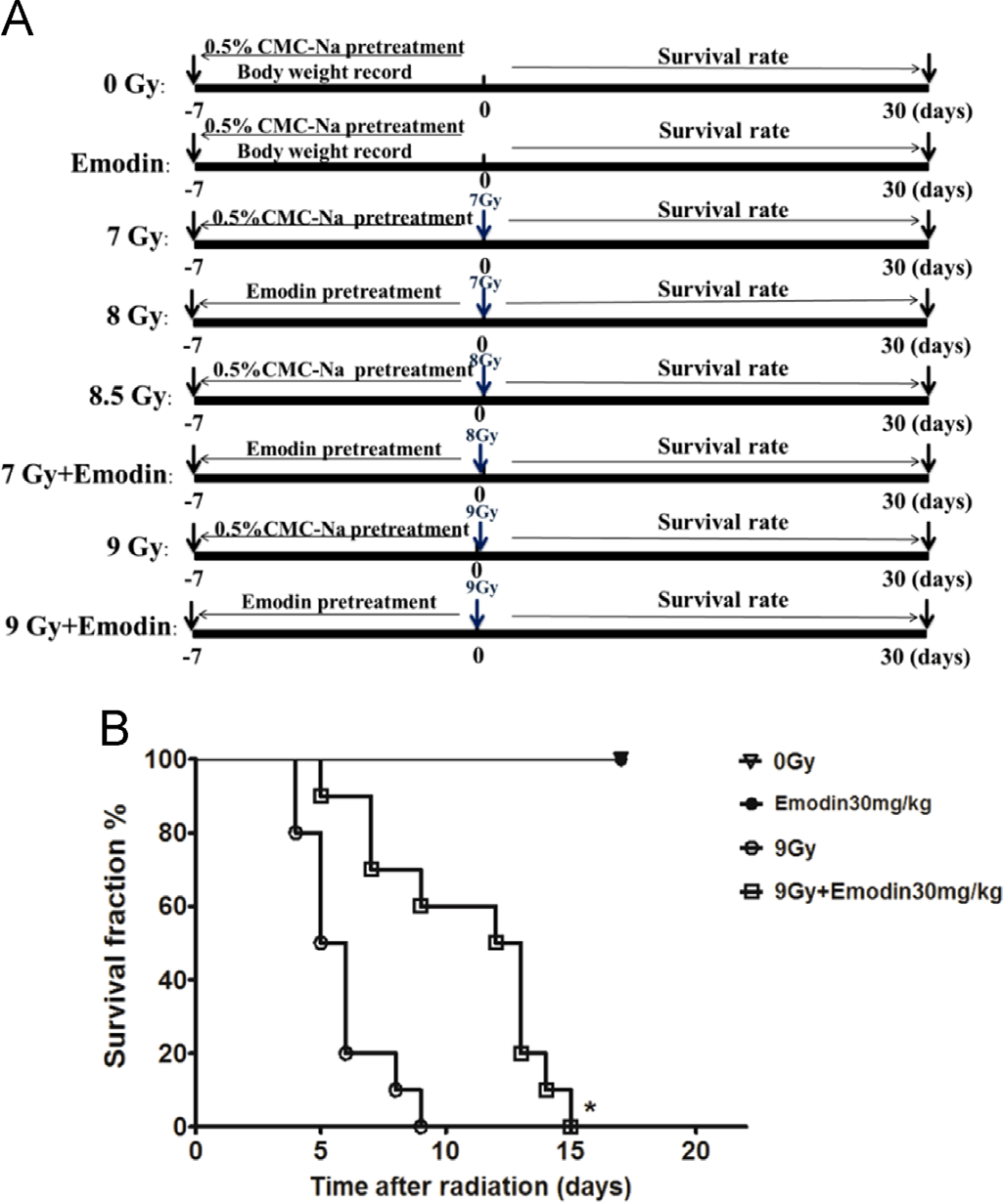
Experimental design schematic picture for animal survival rate (A) and emodin protects mice against radiation-induced death after 9Gy total body irradiation (B). (*n*=10; *p<0.05, emodin treatment group *vs.* radiation group).

**Fig. 2:**
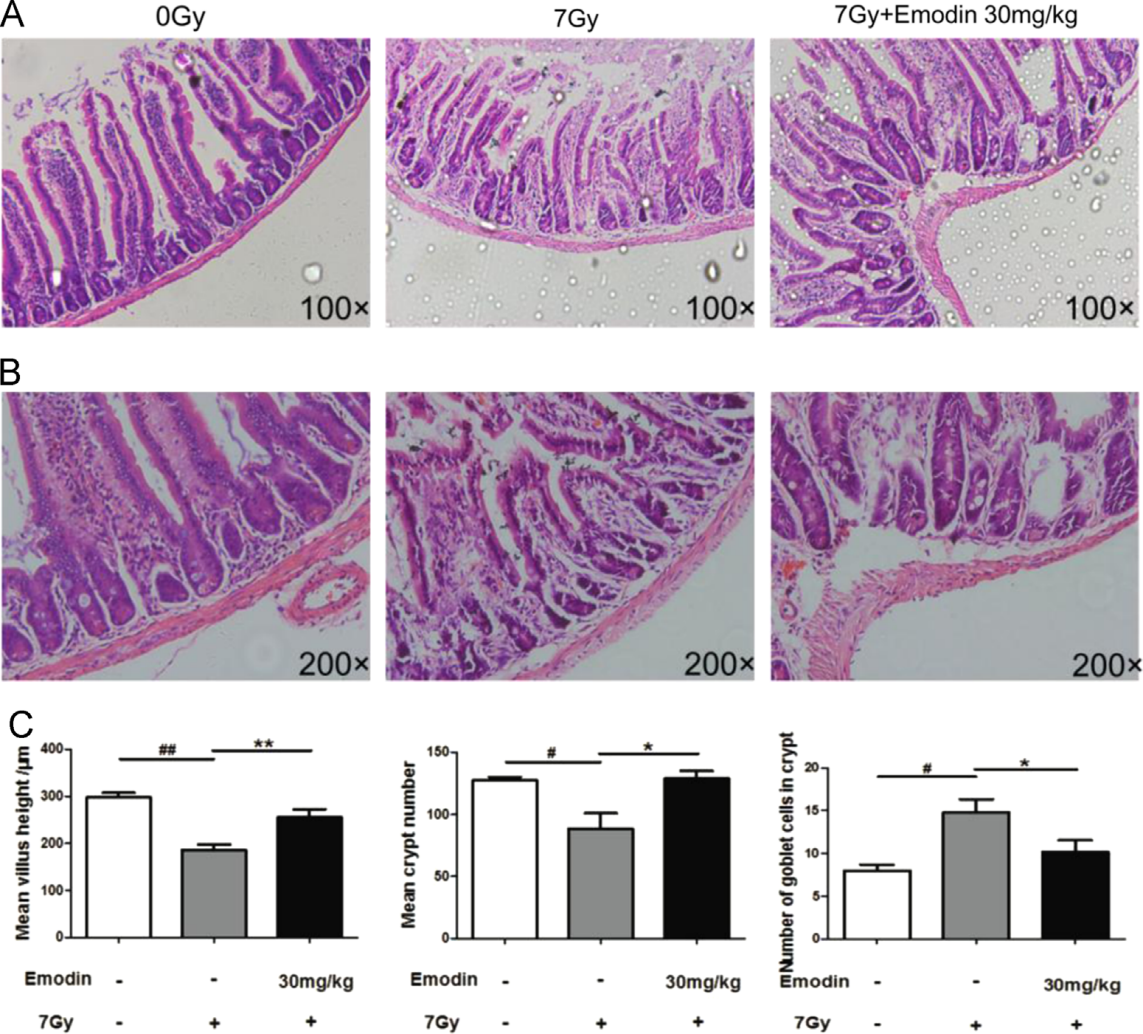
Data on emodin treatment (30 mg/kg/day) attenuate radiation-induced (7Gy) intestinal injury in mice. (A)(B) Representative micrographs of H&E-stained sections of the proximal jejunum. Significant changes were observed emodin treatment attenuated jejunum injury at 7 days post-irradiation. (C) Emodin pretreatment significant increases the villus length and crypts as well as reduces goblet cells number in the irradiated mice at 7 days after 7Gy TBI. (*n*=5; ^#^*p*<0.05, ^##^*p<*0.01, control group *vs.* radiation group; ^*^p<0.05, ^**^p<0.01, emodin treatment group *vs.* radiation group.).

**Fig. 3:**
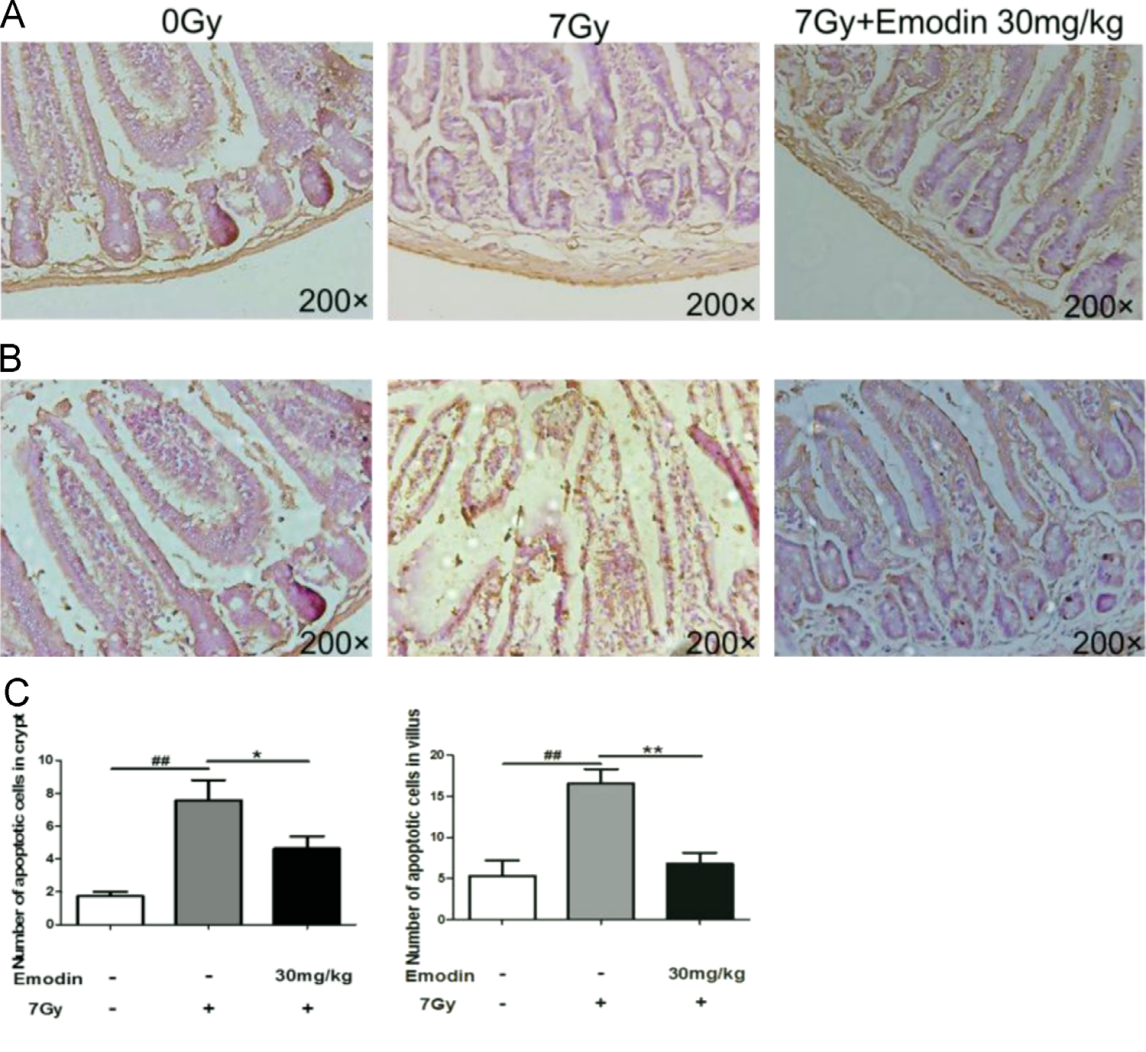
Apoptotic changes in crypt (A) and villi (B) of jejunum tissue. Original magnification 200x.(C)Quantification of TUNEL-positive cells in crypt and villus per group. Values are presented as mean±SEM (*n*=5;^##^*p*<0.01, control group *vs.*radiation group;^*^p<0.05,^**^p<0.01,emodin treatment group *vs.* radiation group.).

**Fig. 4:**
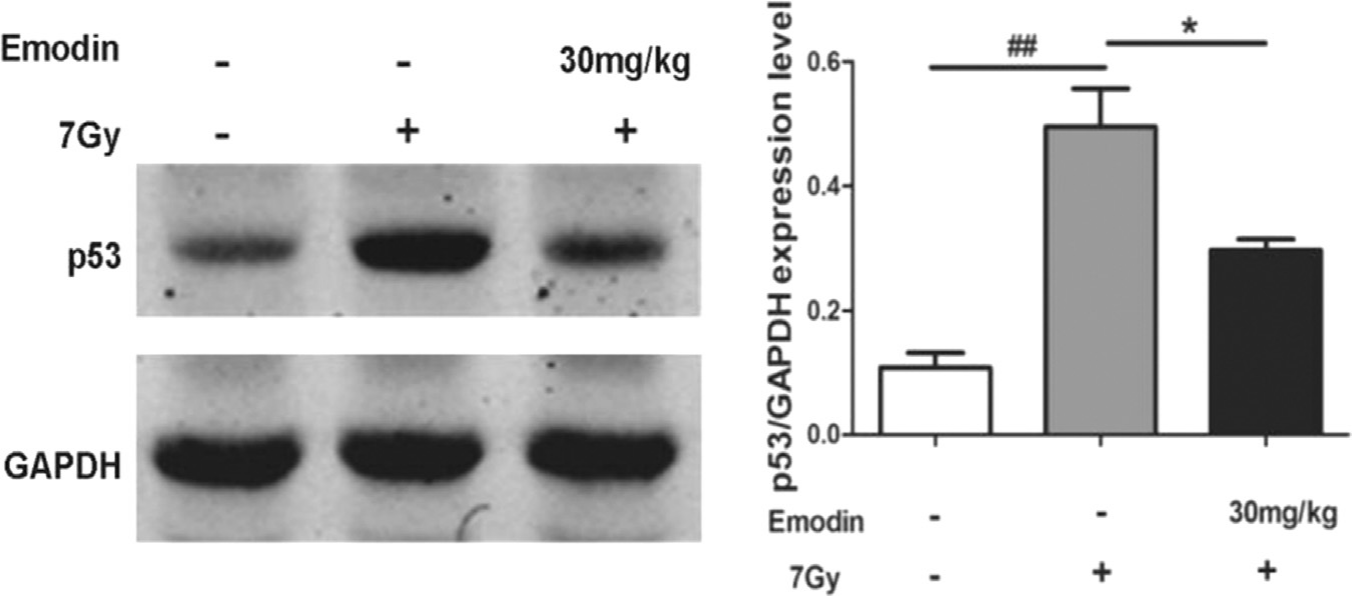
Data on emodin suppresses the radiation-induced expression of p53 for 7 days in the small intestine. Values are presented as the mean±SEM. (*n*=5; ^##^*p*<0.01*,* control group *vs.*radiation group; *p<0.05*,* emodin treatment group *vs.* radiation group).

**Fig. 5:**
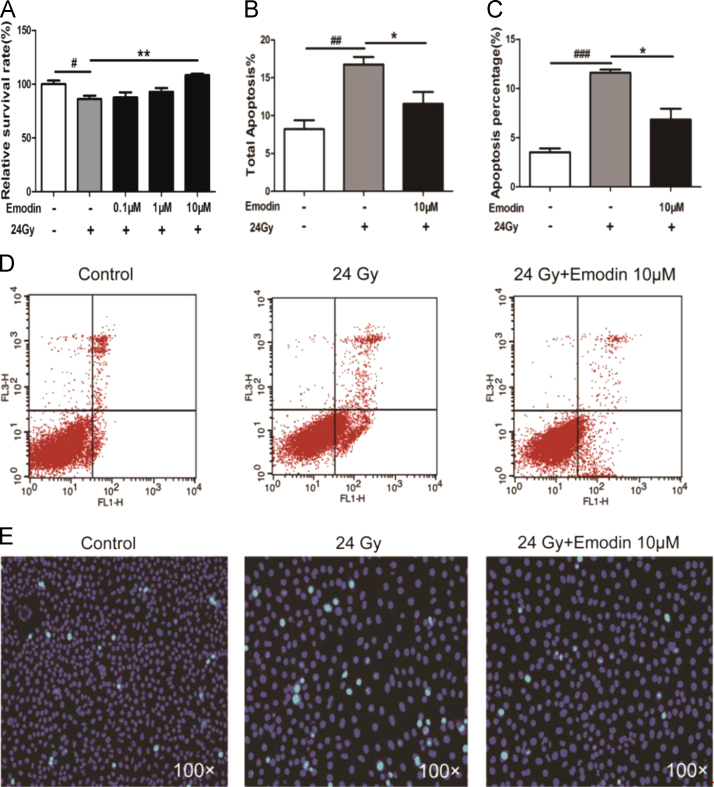
Data on Emodin increased cell viability and reduced apoptosis of HUVECs after gamma irradiation (24Gy). (A)Improvement in the HUVECs survival rate after irradiation in the emodin-treated group (10, 1, and 0.1 µM). (B)and(D) Annexin V/PI double staining assessed using flow cytometry. (C)and(E)Hoechst 33258 staining was performed to evaluate apoptosis in the HUVECs. Emodin was added at 24 h prior to exposure to radiation, then both cell viability and cell apoptosis were analyzed at 24 h post-irradiation. Values are presented as the mean±SEM (*n*=3;^*#*^*p*<0.05,^*##*^*p*<0.01, ^*###*^*p*<0.001*,*control group *vs.* radiation group; ^*^p<0.05,^**^p<0.01, emodin treatment group *vs .*radiation group).

The data showed associated information on emodin against radiation induced mortality (Fig. 1), intestinal injury (Fig. 2, Fig. 3, Fig. 4), apoptosis (Fig. 3) and expression of p53 gene (Fig. 5).

## 2 Experimental design, materials and methods

### 2.1 Experimental design and materials

Male C57BL/6 mice were purchased from SIPPR-BK Experiment Animal Co. (Shanghai, China). Human umbilical vein endothelial cells (HUVECs) from ALLCells, LLC (Emeryville, CA, USA). Emodin (purity ≥98%) was purchased from Dalian Meilun Biology Technology CO. (Dalian, China).

### 2.2 Method

Mice were randomly divided into different groups, and treated different groups with according to the experiment design. The mice survival rates of mice were calculated using the Kaplan–Meier method, then H&E staining, TUNEL staining and tissue Western blot assay were used to analysis the mice intestinal injury [1], [2]. To assess the effect of emodin on HUVECs *in vitro*, using different concentration emodin to treat HUVECs. Using MTT assay, flow cytometry, Hoechst 33258 staining,real-time PCR and Western blot to detect the changes in cell viability,apoptosis,gene and protein [3].
