# Supplementary material for: Data on the radioprotective effect of emodin in vivo and vitro via inhibition of apoptosis and modulation of p53
Source: Data Brief. 2016 Dec 30;11:290–5. doi: 10.1016/j.dib.2016.12.038 (PMC5328685; doi:10.1016/j.dib.2016.12.038)
Supplement: Supplementary file 1 — Supplementary material [file mmc1.docx]

**Conflict of Interest Statement**

We declare that we have no financial and personal relationships with other people or organizations that can inappropriately influence our work, there is no professional or other personal interest of any nature or kind in any product, service and / or company that could be construed as influencing the position presented in, or the review of, the manuscript entitled “*Data on the radioprotective effect of Emodin in vivo and vitro via inhibition of apoptosis and modulation of p53*”.

**Authors:** Jing Wang^b,1^, Yue Zhang^b,1^, QiuzhenZhu^a^, YulanLiu^a^, HaoCheng^b^, YuefanZhang^a,*^, Tiejun Li ^a,*^

**Affiliations:**

**^a^** Department of Pharmacology, College of Pharmacy, Second Military Medical University, Shanghai 200433, China

**^b^** College of Pharmacology, Anhui University of Chinese Medicine, Hefei, Anhui 230012, China
